#  Studying the Stability of S-Layer Protein of *Lactobacillus Acidophilus* ATCC 4356 in Simulated Gastrointestinal Fluids Using SDS-PAGE and Circular Dichroism 

**Published:** 2013

**Authors:** Neda Eslami, Rouha Kasra Kermanshahi, Mohammad Erfan

**Affiliations:** a *Department of Pharmaceutics, School of Pharmacy, Shahid Beheshti University of Medical Sciences, Tehran, Iran.*; b *Department of Biology, School of Sciences, Alzahra University of Basic Sciences, Tehran, Iran*.

**Keywords:** S-layer protein, *Lactobacillus acidophilus *ATCC4356, Stability, Gel electrophoresis, Circular dichroism

## Abstract

Crystalline arrays of proteinaceous subunits forming surface layers (S-layers) are now recognized as one of the most common outermost cell envelope components of prokaryotic organisms. The surface layer protein of *Lactobacillus acidophilus *ATCC4356 is composed of a single species of protein of apparent molecular weight of 43-46 KDa. Considering the *Lactobacillus acidophilus *ATCC4356 having the S-layer is stable in harsh gastrointestinal (GI) conditions, a protective role against destructive GI factors which has been proposed for these nanostructures. It opens interesting perspectives in the using and development of this S-layer as a protective coat for oral administration of unstable drug nanocarriers. To achieve this goal, it is necessary to study the *in-vitro *stability of the S-layers in the simulated gastrointestinal fluids (SGIF). This study was planned to evaluate the *in-vitro *stability of the extracted S-layer protein of *Lactobacillus acidophilus *ATCC4356 in SGIF using it as a protective coat in oral drug delivery. Sodium dodecyl sulfate gel electrophoresis (SDS-PAGE) and circular dichroism (CD) spectroscopy were used to study the stability of the S-layer protein incubated in SGIF. Both the SDS-PAGE and CD spectra results showed that *Lactobacillus acidophilus *ATCC4356 S-layer protein is stable in simulated gastric fluid (SGF) with pH = 2 up to 5 min. It is stable in SGF pH = 3.2 and above it, with and without pepsin. It is also stable in all the simulated intestinal fluids. This S-layer is also stable in all of the simulated intestinal fluids.

## Introduction

One of the most commonly observed surface structures on the prokaryotic cell envelopes is monomolecular crystalline array of proteinaceous subunits with oblique, square and hexagonal structures termed Surface Layers or S-layers. S-layer monomers can be detached from underlying cell wall polymer and from each other using high concentration of Urea, Guanidine hydrochloride and Lithium chloride (1-5). 

S-layer proteins from Lactobacilli have some similarities with other S-layer proteins in terms of the high percentage of hydroxylated and hydrophobic amino acid residues and the low content of sulfur containing amino acids. One of the specific characteristics of S-layer proteins of lactobacilli is the high content of positively charged residues which makes them highly basic protein with isoelectric point ranging from 9.4 to 10.4 and their small molecular mass ranging from 25 KDa to 71 KDa, which is very different from the rest of S-layer proteins, more acidic and with molecular masses up to 200kDa. S-layer protein of *Lactobacillus acidophilus *ATCC4356 is composed of a single, non-glycosylated protein of around 45 KDa (6-8).

Having small size and being resistant against harsh conditions (low pH, high temperature and pressure and enzymes), S-layer protein of *Lactobacillus acidophilus *ATCC4356 is an ideal candidate for serving as a protective coat for oral drug delivery carriers. In this sense, it is essential to evaluate its stability in gastrointestinal (GI) conditions.

Sodium dodecyl sulfate poly acryl amide gel electrophoresis (SDS- PAGE) has been used as an important tool for investigating the *in-vitro *stability of different proteins in simulated gastrointestinal conditions (9-16). Circular dichroism (CD) spectra have been used to analyze acid, heat, enzymes and chemical-induced unfolding (17-20).

In the present work, the S-layer protein of *Lactobacillus acidophilus *ATCC 4356 was extracted with three different chemical treatments and the extraction yields were compared on the basis of statistic tests. SDS-PAGE was used for the primary analysis of S-layer protein stability in the simulated gastrointestinal fluids (SGIF).

The unfolding process of the S-layer protein incubated in SGIF analyzed via CD provided a measure of secondary structural stability in SGIF.

## Experimental

*Bacterial strain and growth condition*

*Lactobacillus acidophilus *ATCC 4356 and *Lactobacillus casei *ATCC393 (as a negative control for isolation of S-layer protein) were obtained from the Persian type culture collection. For the purpose of cultivation, the lyophilized bacteria were reactivated in MRS broth (Merck, Germany), with pH = 6.5 at 37°C overnight. The fresh activated *Lactobacillus acidophilus *ATCC4356 was used for the extraction of S-layer protein.

*Extraction of the S-layer proteins*

*Extraction of S-layer protein with 4M GHCL*

For extraction of *Lactobacillus acidophilus *ATCC4356 S-layer protein, the bacteria were harvested at the end of log phase (with optical density of 0.7 at 695 nm) by centrifugation LK 90 Optima (Beckman Coulter, USA) (at 15000 ×g for 15 min at 4°C) and washed twice with chilled distilled water. The end log harvested cell pellets were treated with 4M GHCL in 50mM Tris-HCl buffer (pH = 7.2), (1 g of harvested cell pellets was suspended in 10-15 mL of 4M GHCl) for 1 h at 37°C. The extracted S-layer protein was separated from the cell pellets by centrifugation (18000 ×g, 15 min, 4°C). The supernatant containing the S-layer protein was dialyzed over night at 4°C against two liters of 50mM Tris- buffer (pH = 7.2) with three times exchange of medium to remove residual GHCl. The extracted S-layer protein was stored at 4°C in 0.02% Na3N for further use (21, 22).

*Extraction of S-layer protein with 1, 5 M LiCl*

Ten to fifteen mg of the end log phase harvested cell pellets were suspended per one milliliter of 1, 5 M LiCl separately, kept at 0°C for 15 min, followed by centrifugation (30000×g, 15 min, 4°C). Both LiCl extracts were dialyzed against two liters of distilled water over night at 4°C with three times exchange of medium to remove residual LiCl. The exracted S-layer protein was stored at 4°C in 0.02% Na3N for further use (23-25).

*Extraction of S-layer protein with 8M Urea (Mechanical extraction)*

The washed harvested cell bacteria at the end of log phase were resuspended 1:1 in a buffer solution of 50mMTris-HCl and 3mM Na3N, pH = 7.2 (standard buffer) . In order to avoid DNA and RNA contamination in the S-protein extraction steps, a few crystals of DNase II and RNase were added. The cells were broken using a high shear fluid processor (M-110S Micro fluidizer processor, Newton, MA) at 4°C, a pressure of 960 bar, and ten passes, which resulted in a complete disintegration. After washing the cell wall fragments two times in standard buffer, the plasma membranes were solubilized in 1%Triton X-100 in standard buffer for 10 min at room temperature. The remaining cell wall fragments were washed twice. Peptidoglycan was lysed by incubating the sample in a standard buffer containing 0.2 mg/mL lysozyme for 6 h at 30°C. The S-layer protein suspension was mixed with 8 M Urea in 50 mM Tris-HCl buffer and pH of 7.4, until the solution became clear. After stirring the solution for 2 h at room temperature, non-protein components were precipitated through centrifugation (12400×g, 60 min and 4°C). The supernatant was dialyzed against two liters 10 mM CaCl2, 3 mM Na3N for 24hr at 4°C. The extracted S-layer protein was stored for further use (26, 27).

*Protein assay*

Surface layer protein concentrations, extracted under different extraction conditions, were determined by Bradford protein assay protocol using bovine serum albumin as a standard (28).

*Sodium dodecyl sulfate poly acryl amide gel electrophoresis (SDS-PAGE):*

Gel electrophoresis of S-layer proteins was performed using 12% (w/v) resolving gel and 4% stacking gel as described previously by Loral *et al. *(29). Molecular mass standard proteins were obtained from Sigma and Cinnagen Company. The gels were run for about 240 min at 150 V, washed with distilled water, stained with Coomassie blue G250 and destained by washing it several times with distilled water.

*UV spectrophotometry*

In order to find the maximum absorbance of the extracted S-layer protein with three different methods, the diluted protein samples were analyzed on RAY LEIGH UV 2601 spectrophotometer between λ of 220 nm and λ of 400 nm. The data were collected using the UV software. The experiments were done triplicate (n = 3).

*Gel electrophoresis evaluation of in-vitro stability of the extracted S-layer protein in simulated gastrointestinal fluid (SGIF)*

Considering the stability of S-layer proteins in harsh conditions such as pH, temperature, proteolysis of some kind and high pressures in one side and suitable small size of *Lactobacillus *surface layers as a protection coat in oral delivery on the other side, it would be necessary to determine S-layer digestion stability facing harsh conditions of gastrointestinal tract. The analytical tool generally used to track the digestion of substrate protein in simulated gastro intestinal fluid is sodium dodecyl sulfate poly acryl amide gel electrophoresis (SDS-PAGE).

Simulated gastrointestinal fluids were prepared as described in the United States pharmacopeia (USP2000) (30). The simulated gastric fluid (SGF) consisted of 2 g/l of sodium chloride (Merck, Germany), containing 3 g/l of pepsin from porcine stomach mucosa, 800- 2500 units/mg, (Sigma-Aldrich, Germany) with pH adjusted to 2 and 3.2 with 37% HCl (30).

The simulated intestinal fluid (SIF) consisted of 6.8 g/L of phosphate potassium monobasic. The pH was adjusted to 6.8 using 0.2 N sodium hydroxide or 0.2N HCl (30).

Fasted state simulated intestinal fluid (FaSSIF) consisted of 3.9 g/L phosphate potassium monobasic, 2.3 g/L sodium taurocholate, 0.56g/l lecithin and 1.1 g/L potassium chloride. The related pH level was adjusted to 6.5 using 0.2N sodium hydroxide (31).

Fed state simulated intestinal fluid (FeSSIF) consisted of 8.24 mL/L acetic acid, 10.2 g potassium chloride, 11.5 g/L sodium taurocholate and 2.8g/L lecithin. The pH level was adjusted to 5 using 0.2 N sodium hydroxide (31).

*In-vitro stability assay of S-layer protein in SGF*

In order to investigate the stability of surface layer protein of *Lactobacillus acidophilus *ATCC 4356 in gastric conditions, three types of SGF were prepared including SGF adjusted to pH = 2 without pepsin and SGF adjusted to pH = 3.2 with and without pepsin.

SGF (1900 μL) was incubated at 37°C for 5 min before addition of 100 μL of S-layer protein (5 mg/mL) at time zero. An aliquot (100 μL) of the digest was withdrawn at different time intervals (0, 5, 15, 30, 60, 90 and 120 min) and was immediately terminated by addition of 30μl of 200mM Na2Co3 and 25 μL of sample buffer. The samples were heated at 100°C for 10 min and analyzed by SDS-PAGE.

*In-vitro stability assay of S-layer protein in SIF*

SIF, FaSSIF and FeSSIF were prepared as previously described. The amount of S-layer protein and the volume of media for protein incubation are the same as that of previous section. An aliquot (100 μL) of the digests was withdrawn at 0 and 240 min of incubation in the mentioned media and added to a separate sampling tube containing 25 μL sample buffer. The tubes were boiled at 100°C for 10 min and the contents were subjected to SDS-PAGE gel.

*Evaluation of the secondary structural changes of the S-layer protein incubated in SGIF by Circular Dichroism (CD)*

Circular Dichroism (CD) is a valuable spectroscopic technique for studying the changes of protein structure in solution because many common conformational motifs, including α-helixes, β-sheets and turns which possess their own distinctive characteristics in Far-UV (200 -250 nm) CD spectra (17-20).

In order to investigate the secondary structural changes of S-layer protein of *Lactobacillus acidophilus *ATCC4356 under various experimental conditions, CD measurements were performed using Jasco- 815 Spectropolarimeter (Jasco Corporation, Tokyo, Japan) at room temperature. Far- UV spectra (200-250 nm) were recorded in a 1 mm -path- length cell, with scan speed of 50 nm/min in a continuous mode. Bandwidth of 2 nm was used. The S-layer protein concentration of 0.25 mg/mL was prepared in different simulated media and conditions. The appropriate buffer spectrum was subtracted from each protein absorbance spectrum and the data were normally plotted as mean residue weight ellipticity versus wavelength. For each sample, five scans were accumulated and averaged.

## Results and Discussion

*Comparison of different extraction S-layer protein methods*

Whole cells of *Lactobacillus acidophilus *were treated in different incubation conditions to extract surface layer protein. The amount of protein isolated by each method was assayed by Bradford protein assay test with the results listed in Table 1.

**Table 1:** Extraction of *Lactobacillus acidophilus *ATCC4356 S-layer protein from whole cell bacteria in different incubation conditions.

Incubation condition of *L. acidophilus*	S-layer protein extraction agent	S-layer protein extracted (mg/mL) (mean ± SEM*, n = 3)	% CV
MRS broth (pH = 6.5) , 37°C in anaerobic jar	GHCl (4M)	0.773 **± **0.012	2.75
MRS broth (pH = 5) , 37°C in anaerobic jar	GHCl (4M)	0.816 **± **0.011	2.47
MRS broth (pH = 6.5) , 37°C in Co2 incubator	GHCl (4M)	0.512 **± **0.006	2.19
MRS broth (pH = 5) , 37°C in Co2 incubator	GHCl (4M)	0.607 **± **0.004	1.23
MRS broth (pH = 6.5) , 45°C in anaerobic jar	GHCl (4M)	0.984 **± **0.004	0.76
MRS broth (pH = 5) , 45°C in anaerobic jar	GHCl (4M)	1.15 ± 0.032	4.93
MRS broth (pH = 6.5) , 45°C in CO_2 _incubator	GHCl (4M)	0.835 ± 0.008	1.44
MRS broth (pH = 5) , 45°C in CO_2 _incubator	GHCl (4M)	1.007 ± 0.001	2.49
MRS broth (pH = 6.5) , 37°C in anaerobic jar	Urea (8M)	0.227 ± 0.006	5.13
MRS broth (pH = 5) , 45°C in anaerobic jar	Urea (8M)	0.354 ± 0.007	3.57
MRS broth (pH = 6.5) , 37°C in anaerobic jar	LiCl (1M)	0.088 ± 0.004	8.64
MRS broth (pH = 5) , 45°C in anaerobic jar	LiCl (1M)	0.111± 0.002	3.39
MRS broth (pH = 6.5) , 37°C in anaerobic jar	LiCl (5M)	0.135 ±0.008	11.08
MRS broth (pH = 5) , 45°C in anaerobic jar	LiCl (5M)	0.182 ± 0.004	4.11

SEM* represents s standard error of the mean values of results from three independent experiments

 It is possible to extract surface layer protein from whole cell of *Lactobacillus acidophilus *ATCC4356 at a remarkably high concentration with 4M Guanidine hydrochloride compared with other extraction methods. All the extraction procedures were repeated three times. Statistical analysis was performed using SPSS statistics 19 and Excel 2010 software. The amounts of S-layer protein isolated through different methods were compared against each other using Student t-test and one-way ANOVA*. *The value of p < 0.05 was regarded as statistically significant. According to statistical analysis, the amounts of S-layer protein extracted using three extraction methods were significantly different (p-value = 0.000). The amount of S-layer protein extraction significantly increased by incubation of bacteria in acidic medium at 45°C in anaerobic condition (p-value = 0.001).

These findings are in line with a study conducted by Khaleghi *et al. *which investigated the effect of environmental stress on S-layer production in *Lactobacillus acidophilus *ATCC4356 based on *slp*A gene expression (*slp*A is the active S-layer protein encoding gene in normal growth condition) (21). They found that environmental conditions influenced the S-layer protein production and slpA gene expression. They showed that S-layer proteins are preferentially expressed in bacteria incubated in MRS broth medium (pH = 5) at 45°C in anaerobic condition.

*UV spectrophotometry*

**Figure 1:**
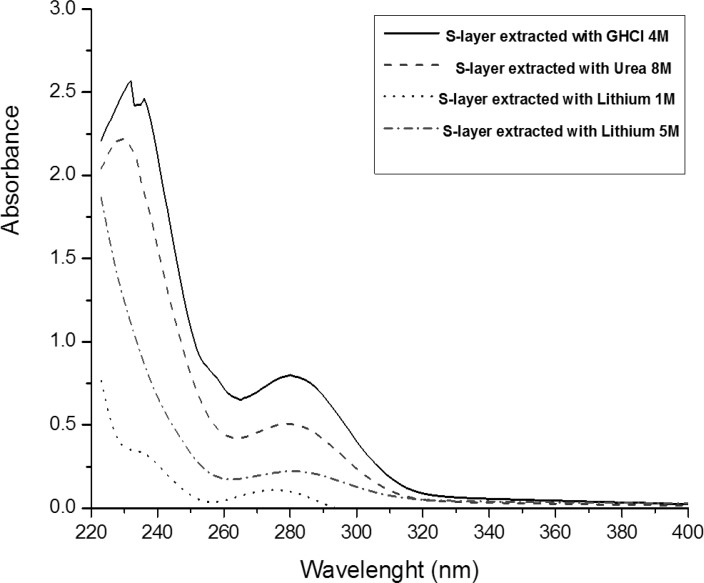
UV spectra of the *Lactobacillus acidophilus *ATCC4356 S-layer protein extracted with three different methods.

Maximum absorbance for all *Lactobacillus acidophilus *ATCC4356 surface layer proteins extracted via different methods was found to be 280nm (λ max = 280 nm). The maximum absorbance is related to GHCl extracted protein (Figure 1). It is in line with the studies on the UV absorbance of LiCl extracted S-layer protein of *Lactobacillus acidophilus *NCC2628 fermented in various media, and maximum UV absorbance of *Bacillus sphaericus *S-layer protein extracted with GHCl (6M) (25, 27).

*In-vitro stability of S-layer protein in simulated gastrointestinal fluids studied with SDS-PAGE*

The present study is the first of its type *in-vitro *S-layer protein stability pattern using gel electrophoresis and circular dichroism. The standardization of the simulating gastrointestinal conditions has been described in the U.S Pharmacopeia. It is not meant to mimic precisely the fate of proteins *in-vivo *conditions but rather to evaluate the susceptibility of the protein to digest under fixed conditions *in-vitro.*

The S-layer protein extracted via different methods revealed a dominant band corresponding to the molecular mass of 43-46 KD (Figure 2). In the first step, the stability of S-layer protein in simulated gastrointestinal fluids was analyzed by SDS-PAGE. It is worth noting that this technique had been formerly used for evaluating the *in-vitro *gastrointestinal stability of different edible proteins by other researchers (10-17).

In the SGF reaction system (pH = 2, without pepsin), the S-layer protein band completely disappeared after 5 min without formation of any digestion fragments (Figure 2a). The low pH of SGF induces the protonation of carboxylic and amine groups in the hydrophilic shell and hence leads to a new distribution of charges and changes in the S-layer protein structure.

In the SGF reaction system (pH = 3.2), whether with or without pepsin, the original S-layer band persisted until the end of the study (120 min) (Figure 2b, 2c), suggesting that SGF with pH = 3.2 and above it, and also pepsin are ineffective in the degradation of the S-layer protein.

This result is similar to former results found in acidic degradation of *Bacillus sphaericus *surface layer protein studied by AFM (32). Tocca Herrera *et al*. have shown that due to exposure of the acidic condition below pH = 3, the original S-layer lattice of *Bacillus sphaericus*is deformed and the typical structure disappeared (32).

**Figure 2:**
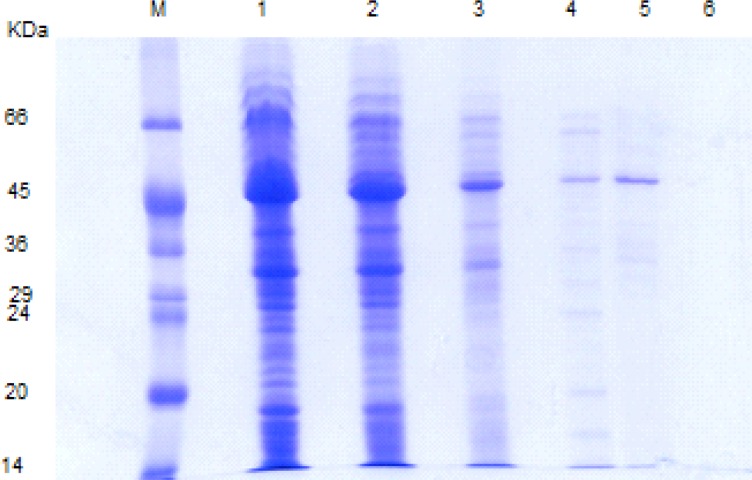
SDS-PAGE pattern of *L .acidophilus *ATCC 4356 S-layer protein extracted via different methods. M: molecular weight marker, Lane1, 2: S-layer protein extracted with GHCl (4M) in water and GHCl (4M) in 50 mM Tris-HCl buffer, lane 3: S-protein extracted with Urea (8M), lane 4, 5: S-protein extracted with LiCl (1, 5 M) , lane 6: Lactobacillus casei without S-layer protein as a negative control

**Figure 2a:**
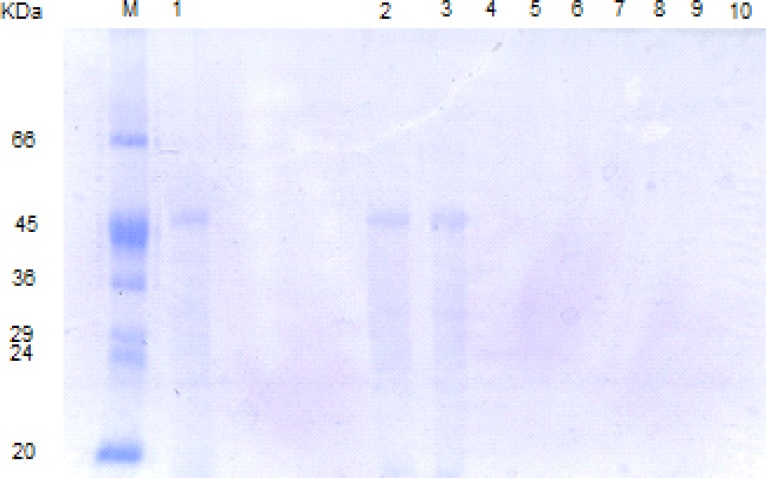
SDS-PAGE patterns of *L. acidophilus *ATCC4356 S-layer protein digested by SGF (pH 2, without pepsin). M: molecular weight marker, Lane 1: Native S-layer protein, lanes 2- 10: SGF digestion pattern of S-layer protein at time = 0, 5, 10, 15, 30,45,60,90,120 min.

**Figure 2b:**
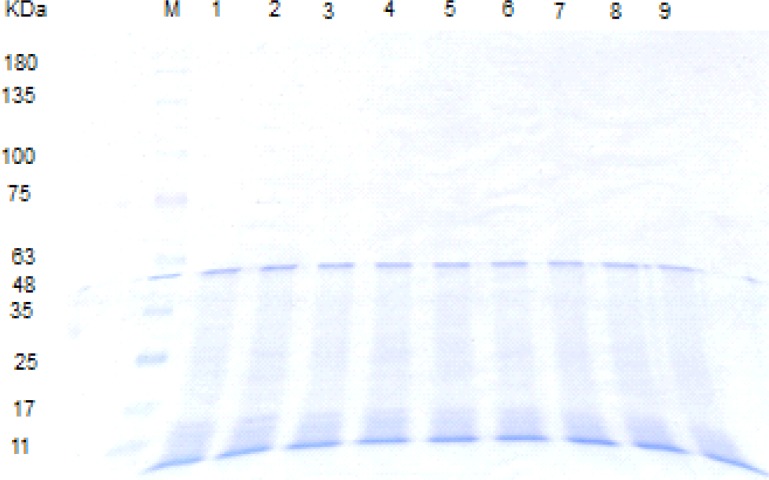
SDS-PAGE patterns of *L. acidophilus *ATCC4356 S-layer protein digested by SGF (pH 3.2, without pepsin). M: molecular weight marker, Lanes 1-8: SGF digestion pattern of S-layer protein at time = 0, 5, 15, 30, 45, 60, 90,120 min.

**Figure 2c:**
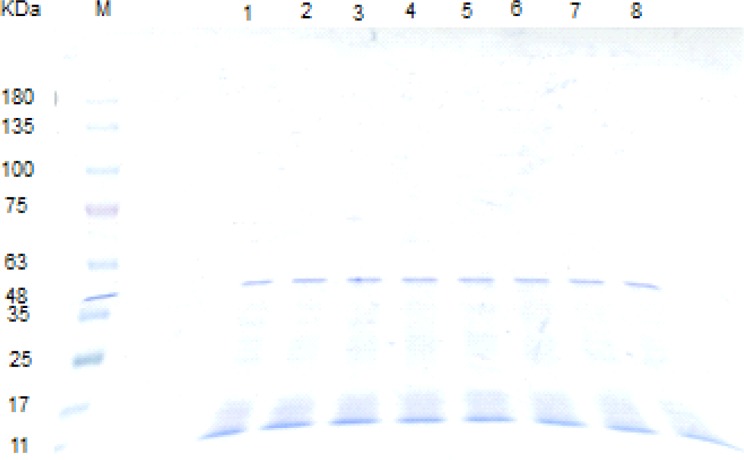
SDS-PAGE patterns of *L. acidophilus *ATCC4356 S-layer protein digested by SGF (pH 3.2, with pepsin). M: molecular weight marker, Lane 1: Native S-layer protein, Lanes 2-9: SGF digestion pattern of S-layer protein at time = 0, 5, 15, 30,45,60,90,120 min.

**Figure 2d:**
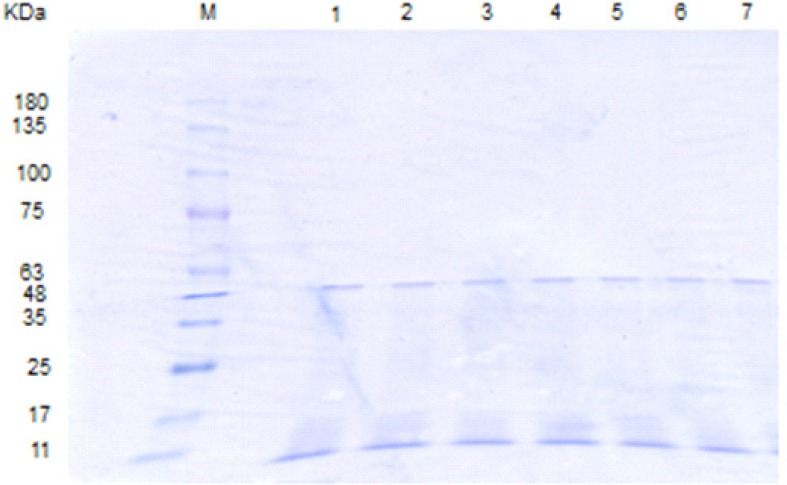
SDS-PAGE patterns of *L. acidophilus *ATCC4356 S-layer protein digested by SIF, FaSSIF and FeSSIF. M: molecular weight marker, Lane 1: Native S-layer protein, lanes 2, 3: S-layer protein digested by SIF at time = 0, 240 min. lanes 4, 5: S-layer protein digested by FaSSIF at time = 0, 240 min. lanes 6, 7: S-layer protein digested by FeSSIF at time = 0, 240 min.

Previous researches concerning the SGF digestion of other edible proteins with approximately similar molecular weights have shown that most proteins are unstable in SGF with very acidic pH. Teshima *et al*. have studied the *in-vitro *gastrointestinal stability of bovine serum albumin (BSA), ovalbumin (OVA), horse radish protein (HRP), beta lactoglubin (BLG) with molecular weights of 63.1, 45.9, 48.8 and 17.7 KDa respectively. Based on it, in SGF (pH 2), BSA is rapidly digested before 1 min, while BLG remains indigested for 60 min. The original band of OVA rapidly decreases but a fragment remains until the end of the study (60 min). HRP is relatively stable in this medium (10).

In our study, the digestion patterns of S-layer protein in SIF, FaSSIF and FeSSIF were quite similar (Figure 2d). The S-layer protein was resistant in all of the above simulated intestinal fluids and the original band could be detected until the final time point (240 min). That is while Teshima *et al*. have found that BSA, OVA and HRP were resistant in SIF condition. In contrast, BLG was easily digested in SIF (10).

*Secondary structural stability of S-layer protein in simulated gastrointestinal fluids studied with CD*

The application of CD to the study of protein folding has been reviewed by Kelly and Prince (33). In order to see whether the conformational changes of S-layer protein occurred under different conditions, Far- UV CD spectra were taken at 25°C by using Jasco 815 spectropolarimeter. Alterations in the secondary structure are measured in the region of 200–250 nm, the so-called Far-UV CD. This region is dominated by contributions of the peptide bonds, although some side chains may also be involved. α- helices display large CD bands with negative ellipticity at 222 and 208 nm and positive ellipticity at 193 nm. *β*-sheets exhibit a broad negative band near 218 nm and a large positive band near 195 nm while disordered extended chains have a weak broad positive CD band near 217 nm and a large negative band near 200 nm. The spectrum of a protein is basically the sum of the spectra of its conformational elements, and thus CD can be used to estimate secondary structure. 

The CD spectrum of fresh extracted *Lactobacillus acidophilus *ATCC4356 S-layer protein in Tris-HCl buffer in the Far- UV (200-250 nm) showed ellipticity with relatively large intensity between 210 and 230 nm, being characteristic of the *α/ β *conformation.

**Figure 3:**
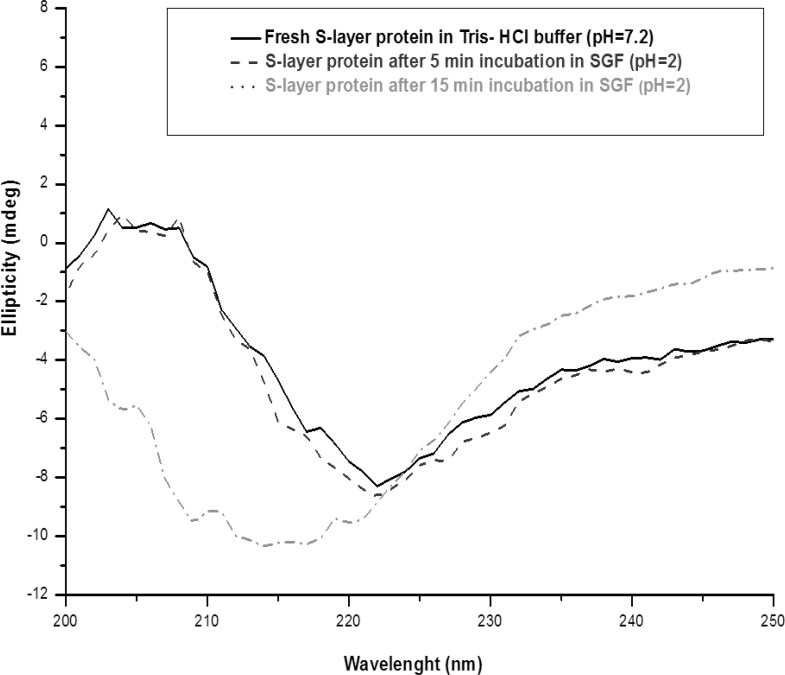
Far-UV CD spectra of S-layer protein of *L.acidophillus*ATCC4356 at 25°C: Fresh S-layer protein in Tris buffer (solid line), S-layer protein after 5 min incubation in SGF (pH = 2) (dash line) and S-layer protein after 15 min incubation in SGF (pH = 2) (dot line). Each spectrum represents the average of five scans.

Fresh extracted *Lactobacillus acidophilus *ATCC 4356 S-layer contained 26% of alpha-helix, 5% of beta-helix, 33% of turn and 36% of random structures. The CD spectrum of S-layer protein incubated in SGF (pH = 2), up to 5 min, is similar to the CD spectrum of the fresh S-layer protein in Tris-HCl buffer (native spectra). It demonstrates that the S-protein retains its secondary structure in pH = 2 for 5 min. After that, the CD spectra significantly altered relative to that for the native spectra, gained content in β-helical structure (from 5% to 30%) and lost content in all other structures with the shifting of peak maxima to the shorter wavelengths in Far- UV (Figure 3). 

**Figure 4:**
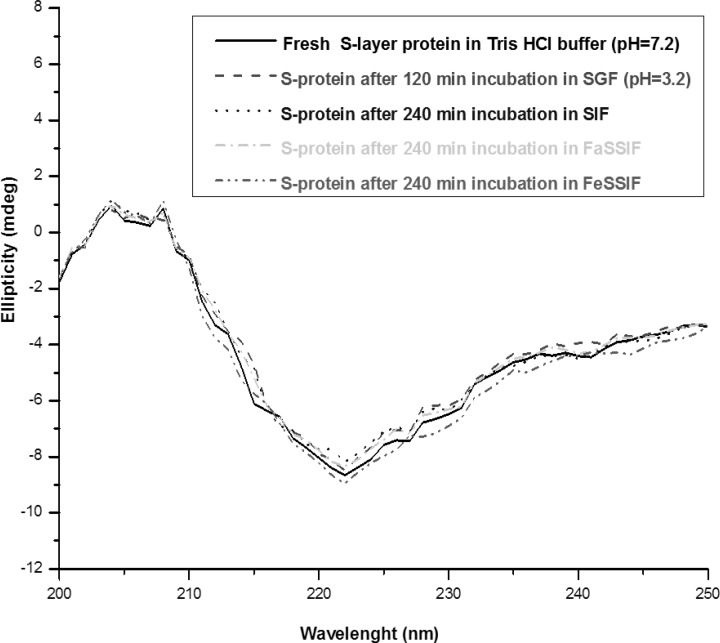
Far-UV CD spectra of S-layer protein of *L. acidophillus *ATCC4356 at 25°C: Fresh S-layer protein in Tris buffer (solid line), S-layer protein after 120 min incubation in SGF (pH = 3.2) (dash line), S-layer protein after 240 min incubation in SIF (dot line), in FaSSIF (dash dot) and in FeSSIF (dash dot dot). Each spectrum represents the average of five scans.

The changes of the secondary structure after 5 min observed with S-layer protein in SGF (pH = 2) can be explained by acid denaturation of the protein. The structure of S-layer protein is pH sensitive and the protein undergoes a structural transition to form a partially unfolded state. Under harsh acidic condition (pH = 2), all of the ionizable protein groups are protonated and the charge repulsion drives the changes of the secondary structure (18, 19, 32). The Far -UV CD spectra of S-layer protein incubated in SGF (pH = 3.2) is similar to the native spectra, which suggests that S-layer protein is stable in that medium (Figure 4).

The Far- UV CD spectras of S-layer protein incubated in SIF, FaSSIF and FeSSIF are superimposable to the native CD spectra, implying that S-layer protein’s secondary structure is also stable in these simulated conditions (Figure 4).

Trust *et al*. investigated the secondary structure changes of S-layer protein of a pathogenic *Aeromonas hydrophila *strain (Mw = 52) under various experimental conditions. The CD spectra of this S-layer protein was incubated in pH = 2 and pH = 7.4, showing that no major conformational changes took place at low pH, implying that the protein was not drastically altered by the low pH (35). In contrast to the instability of *Lactobacillus acidophilus *ATCC 4356 S-layer protein in low pH, the S-layer of pathogenic *Aeromonas hydrophila *strain is stable in this condition.

Alcocer *et al*. measured the digestibility of Sunflower albumin and Brazil nut 2S albumin in simulated gastrointestinal fluids by SDS-PAGE and CD. For each protein, spectra obtained at pH = 2.2, were superimposable to those recorded at 6.8, confirming that these proteins are highly resistant to acidic pH (36).

## Conclusion

S-layers are crystalline monomolecular assemblies of protein or glycoprotein, which represent one of the most common cell surface structures in Bacteria. Using the S-layer proteins as a protective coat in drug delivery and drug targeting, it is necessary to evaluate their *in-vitro *gastrointestinal stability to predict their behavior facing harsh conditions of gastrointestinal system. In this work, the *in-vitro *gastrointestinal stability of *Lactobacillus acidophilus *ATCC 4356 S-layer protein was studied by gel electrophoresis and circular dichroism.

The results confirmed that *Lactobacillus acidophilus *ATCC 4356 S-layer protein is readily digested under simulated gastric fluids (pH = 2) as demonstrated by both SDS-PAGE and CD spectra. It is also confirmed that the S-layer protein is stable in simulated gastric fluids (pH > 3) with and without pepsin and also stable in simulated intestinal fluids in both fasted and fed states. Our results showed that the S-layer protein of *Lactobacillus acidophilus *ATCC 4356 has the potential to be used as a protective coat for unstable oral drug delivery carriers.
